# Assessment of Bronchiectasis in HIV Patients among an Urban Population

**DOI:** 10.1155/2020/8903809

**Published:** 2020-02-27

**Authors:** Veena Dronamraju, Navneet Singh, Justin Poon, Sachi Shah, Joseph Gorga, Hector Ojeda-Martinez, Samy McFarlane

**Affiliations:** ^1^Department of Internal Medicine, SUNY Downstate Medical Center, USA; ^2^Department of Infectious Disease, Montefiore Medical Center, USA; ^3^Division of Pulmonary and Critical Care Medicine, New York Health and Hospitals Corporation, Kings County Hospital Center, USA; ^4^Department of Infectious Disease, SUNY Downstate Medical Center, USA; ^5^Department of Endocrinology, SUNY Downstate Medical Center, USA

## Abstract

Bronchiectasis is characterized by permanent destruction of the airways that presents with productive cough, as well as bronchial wall thickening and luminal dilatation on computed tomographic (CT) scan of the chest; it is associated with high mortality. Accumulating data suggests higher rates of bronchiectasis among the HIV-positive population. This case series involves 14 patients with bronchiectasis and HIV followed at two major urban institutions from 1999 to 2018. Demographics, clinical presentation, microbiology, radiographic imaging, and outcomes were collected and compiled. Mean age was 42 years (range 12-77 years). 36% had a CD4 count greater than 500 cells/mm^3^, 28% had a CD4 count between 200 and 500 cells/mm^3^, and 36% had AIDS. 43% were treated for Pneumocystis jiroveci pneumonia (PJP) and 50% for Mycobacterium avium complex (MAC) infection. 21% had COPD, 7% had asthma, and 7% had a history of pulmonary aspergillosis. Two patients were followed up by pulmonary services after diagnosis of bronchiectasis on CT. The timeline of the follow-up in these cases was within months and after three years respectively. It is posited that the prevalence of bronchiectasis in HIV patients may be underestimated. Improving recognition and management of bronchiectasis could help diminish rehospitalization rates.

## 1. Introduction

Bronchiectasis is a permanent distortion of airways clinically characterized by productive cough and diagnosed with the presence of bronchial wall thickening and luminal dilatation on CT scan of the chest [1, 2]. There is a high prevalence of noncystic fibrosis bronchiectasis associated with underlying chronic lung diseases, rheumatoid arthritis, connective tissue disease, inflammatory bowel disease, and HIV [3]. The disease is difficult to recognize as its clinical features overlap with many other chronic lung diseases [4, 5]. Furthermore, even when the disease is properly identified, there is often inadequate treatment due the lack of understanding of the relationship between the underlying cause and bronchiectasis.

In the United States, there are currently no standardized treatment guidelines for the disease. British Thoracic Society guidelines recommend antibiotics for acute and maintenance therapy [6]. This approach to management presumes infectious origins of the disease, which may not be an accurate presumption. For instance, bronchiectasis in patients with HIV remains apparent despite availability of antiretroviral therapy and subsequent reduction of pulmonary infections.

To further understand the relationship between these two diseases, we present a case series of bronchiectasis in HIV-positive patients seen at two major urban institutions.

## 2. Subjects and Methods

A search of the electronic medical records at Kings County Hospital Center and SUNY Downstate Medical Center was completed looking for patients with both bronchiectasis and HIV infection identified by either ICD-9 (042 for HIV and 494.0 for bronchiectasis) or ICD-10 codes (B20 for HIV and J47 for bronchiectasis). Of approximately 3000 patients, 14 patients with both diagnoses were identified from 1999 to 2018. Demographics, clinical characteristics, diagnostic imaging, microbiology, and clinical course were obtained and are summarized in Tables 1 and 2.

### 2.1. Case Descriptions

#### 2.1.1. Patient 1

A 12-year-old female with congenitally acquired HIV and a known history of bronchiectasis per patient survey presented with a productive cough that was treated with azithromycin. One year later, the patient reported purulent cough with rhinorrhea and nasal congestion. The patient had a repeat infection the next year, in which she had rhinorrhea and nasal congestion and completed a ten day course of amoxicillin. The patient moved to North Carolina later the same year and is presumed to be following there. Patient had no confirmatory CT scans obtained during her course.

#### 2.1.2. Patient 2

A 17-year-old female with congenital AIDS arrived from Haiti with symptoms of productive blood-streaked cough, rhinorrhea, and chest pains. The patient was hospitalized for pneumonia and was found to have evidence of bronchiectasis and air-filled bronchiectatic cysts in the left lower lobe on CT scan. Her previous history was significant for being hospitalized for six months because of a “lung problem” three years prior in Haiti. The patient presented to the chest clinic shortly after her initial hospitalization with another bout of dyspnea on exertion; she was started on albuterol treatments, which improved her symptoms. In the following year, the patient presented with cough productive of white sputum, fevers, and myalgias. She was subsequently admitted for a presumed exacerbation of bronchiectasis with PJP and viral syndrome on the differential. In the next two years, the patient was admitted several times for various infectious syndromes, including esophageal candidiasis, upper respiratory viral infection, pyelonephritis, and facial cellulitis.

#### 2.1.3. Patient 3

A 19-year-old female with congenital AIDS was being followed in the pediatric immunology clinic for routine care. Over several years, she was treated for what was presumed to be recurrent bacterial sinusitis with symptoms of a dry cough and rhinorrhea with no fevers. She was prescribed several courses of amoxicillin/clavulanate. One year later, she was treated for oropharyngeal candidiasis several times and twice for esophageal candidiasis. During one of these admissions, she had a CT scan of the chest that demonstrated RLL bronchiectasis with scattered pneumatoceles. The next year, she was treated for otitis and then presumptively for PJP. The following year, she was admitted for anemia thought to be due to possible parvovirus in the background of a MAC infection. She was given IVIG and treated with anti-infectives for MAC. In subsequent years, she was treated two more times for presumed PJP (sputum negative) and MAC (sputum positive). This patient had several repeat chest CT scans over the course of her clinical picture which all remained stable.

#### 2.1.4. Patient 4

A 22-year-old female with congenital AIDS who was intermittently compliant with ART) had a history of multiple hospital admissions for pneumonia, both community-acquired and PJP. Given the recurrence of infection, her primary care physician suspected bronchiectasis, but did not order chest imaging. One year later, the patient was seen in clinic for worsening cough and was treated with amoxicillin/clavulanate for bronchiectasis exacerbation. The patient had six admissions over the next year, one of which was for sepsis and the others for persistent cough in the absence of signs of pneumonia. She underwent multiple courses of antibiotic treatments over this time. This patient did not have diagnostic imaging to confirm her bronchiectasis during her course.

#### 2.1.5. Patient 5

A 25-year-old female with congenital HIV who was noncompliant with ART and had a history of multiple admissions for severe pneumonia was admitted to the medical ICU for severe community-acquired pneumonia. Her hospital course was complicated with Haemophilus influenzae bacteremia and a pleural effusion. Her pleural fluid culture revealed MAC, and her symptoms improved with antibiotics. She was restarted on ART and PJP prophylaxis. During the admission, the patient had a CT scan that confirmed bronchiectasis in the right upper, right middle, and bilateral lower lobes. Sputum culture grew normal flora. The patient was discharged and remained noncompliant with HIV treatment. She did not follow up outpatient. The next year, she had three more admissions during which she received empiric treatment for both community-acquired pneumonia and PJP. The following year, the patient was admitted for severe pneumonia with confirmed PJP along with positive enterovirus and rhinovirus. The patient initially improved, however, she then developed a stroke that was thought to be secondary to disseminated MAC with HIV vasculitis on the differential. Her blood culture grew MAC, and she was treated accordingly. The patient's poor clinical condition persisted, and she decided to suspend treatment and transition to comfort care.

#### 2.1.6. Patient 6

A 35-year-old female with history of congenital HIV and asthma was admitted to the hospital for persistent cough. The patient was diagnosed with likely bronchiectasis exacerbation with pneumonia on the differential. Her symptoms improved with a ten-day course of levofloxacin. Following this admission, the patient had a CT scan that showed bronchiectasis in the right upper, right middle, and bilateral lower lobes. The disease was most severe in the right middle lobe. The patient was never seen in the chest clinic. Of note, this patient had relatively well-controlled HIV, with no admissions related to PJP. She had multiple admissions for asthma in the past and once required intubation.

#### 2.1.7. Patient 7

A 41-year-old female with AIDS who was noncompliant with ART and had a history for several admissions for recurrent pneumonias was admitted for presumed PJP. CT chest showed diffuse ground glass opacities, and bronchoalveolar lavage (BAL) was negative for PJP. The patient was still treated with prednisone and trimethoprim/sulfamethoxazole for presumed PJP. Over the next year, the patient had three additional admissions for respiratory infections. During one of the admissions, the patient had an incidental finding of bulky adenopathy near the superior mesenteric artery. A biopsy of the node showed granulomatous lymphadenitis along with acid fast bacteria consistent with Mycobacterium species. The CT chest also demonstrated bronchiectasis of the right middle lobe and inferior lingula. The patient was discharged with treatment for tuberculous and MAC for one month. She was readmitted due to worsening of symptoms before completion of her treatment. The next year, the patient was admitted and treated for MAC bacteremia. Later that year, she was found to have worsening adenopathy throughout the body that was thought to be due to disseminated MAC. She was again admitted in 2016 for hypoxic respiratory failure with CT scan that showed worsening pleural effusion and tree-in-bud opacities. Broad spectrum antibiotics were initiated for septic shock, and the patient was transferred to the medical ICU where she expired.

#### 2.1.8. Patient 8

A 48-year-old male with hypertension, COPD, and benign prostatic hyperplasia was admitted for weight loss with cough productive of yellow sputum for five weeks. He was diagnosed with pulmonary tuberculosis and HIV, and started on RIPE therapy. I patient clinically improved; however, he continued to have worsening radiographic consolidations that prompted concern for aspergillosis with resistant tuberculosis on the differential. The patient underwent bronchoscopy with bronchoalveolar lavage revealing Aspergillus species. His tuberculosis medications were discontinued, and the patient was started on Voriconazole to treat chronic necrotizing aspergillosis. The next year, the patient underwent right upper lobe wedge resection for persistent symptoms of pulmonary aspergilloma and proceeded to complete a seventeen-month course of Voriconazole. Two years later, the patient developed a chronic cough productive of clear sputum and was treated for an upper respiratory illness in the setting of a COPD exacerbation. An episode of hematuria brought the patient into the emergency department, where he was found to have new right lower lobe opacity caught on CT scan of the abdomen and pelvis. On further history, he was found to have severe cough with low-grade fevers. A CT chest showed interval progression of bronchiectasis and bronchial wall thickening in the right middle lobe and right lower lobe with mucus-filled airways as well as interval development of a peripheral airspace opacity. A repeat CT chest the next year showed widespread bronchiectasis with retained secretions. The patient was started on albuterol, and his symptoms improved. Almost two years later, the patient was diagnosed with poorly differentiated rectal adenocarcinoma and started on FOLFOX with palliative radiation. He was subsequently admitted for hypotension and expired.

#### 2.1.9. Patient 9

A 48-year-old female with AIDS, a history of PJP complicated by pneumothorax, and a history of two admissions for community-acquired pneumonia who was admitted for one month of syncopal episodes. She was treated for MAC based on sputum and blood cultures. Two months later, the patient was admitted to the hospital for sepsis likely secondary to disseminated MAC with PJP on the differential. She was empirically treated for both MAC and PJP. CT scan of the chest was completed during this admission and showed bronchiectasis in right upper, right middle, and bilateral lower lobes. The patient had no further follow-up.

#### 2.1.10. Patient 10

A 54-year-old female with HIV and previously documented bronchiectasis presented for fever, chill, and cough. The patient was admitted for community-acquired pneumonia. CT chest demonstrated that the patient had exacerbation of bronchiectasis with superimposed infection. She was discharged home with a seven-day course of levofloxacin.

#### 2.1.11. Patient 11

A 55-year-old female with HIV and a remote history of tuberculosis was found to have metastatic cervical cancer to the pelvis, liver, and likely lung. Later that year, she was found to have reactivated tuberculosis with MAC on the differential. CT chest showed multiple cystic cavities, predominantly in the upper lobes, along with parenchymal scarring consistent with bronchiectasis. Despite optimal treatment with RIPE and clarithromycin, the patient had worsening hemoptysis secondary to disseminated intravascular coagulation and expired.

#### 2.1.12. Patient 12

A 62-year-old female with history of large B cell lymphoma and HIV was admitted for pneumonia. The patient had a CT chest confirming bronchiectasis in the right upper lobe and lingula. Sputum culture grew Haemophilus influenzae. She clinically improved with antibiotics and was discharged. She subsequently had several admissions for community-acquired pneumonia and did not follow up outpatient.

#### 2.1.13. Patient 13

A 70-year-old male with HIV, history of pulmonary tuberculosis and asthma/COPD presented to the emergency department with complaints of a productive cough and was treated for pharyngitis. Shortly after this episode, the patient was treated for an upper respiratory tract infection as an outpatient. Following a trip to the Dominican Republic, the patient began complaining of intermittent episodes of severe productive cough. Over the next few years, the patient had numerous hospitalizations for multiple recurrent upper respiratory tract infections, right hip cellulitis, and conjunctivitis. Five years after initial admission, the patient was recognized to have chronic bronchiectasis. CT chest showed an air-filled bronchiectatic cyst in the left lower lobe. He was started on azithromycin and advised to take it as needed during future episodes. Two years later, the patient was hospitalized twice, once for acute bronchitis for which he was treated with azithromycin and another time for hemoptysis. Three years after diagnosis of bronchiectasis, the patient was seen in the chest clinic for the first time.

#### 2.1.14. Patient 14

A 77-year-old female with history of HIV was admitted for pneumonia. The patient was hypoxic and chest X-ray showed bilateral hazy opacities. CT scan of the chest showed bilateral mild bronchiectasis at the lung bases. During the admission, the patient had a repeat CT chest which showed radiographic signs suggestive of PJP. She received treatment for PJP empirically with trimethoprim/sulfamethoxazole and prednisone. The patient clinically improved.

## 3. Discussion

Bronchiectasis develops in the context of repeat occurrence of bronchial insult from infection or inflammation [1, 7]. The insult develops from pulmonic, oncologic, rheumatologic, infectious, or multifactorial disease [8–10]. Microbial toxins and increased inflammation compromise mucociliary clearance [9–12] causing patients to become susceptible to microbial colonization [2, 13]. This colonization then propagates further infection and inflammation, which creates a self-perpetuating cycle [14]. Bacterial colonization is therefore associated with increased airway damage and worsened severity of disease [2, 15, 16]. Most commonly, patients are colonized with Haemophilus influenzae and Pseudomonas aeruginosa [2, 13]. A Pseudomonas predominant lung microbiome has been associated with a worse clinical phenotype of bronchiectasis [16–18].

A minimal amount of research has been done in the field regarding bronchiectasis. A recent multisite study conducted under the Bronchiectasis Research Registry used a large sample size to characterize different aspects of the disease, identify prevalent medical comorbidities, and develop a microbiome of the lung in bronchiectasis [19]. Review of previous smaller-scale studies that focus on fewer aspects has shown differences in the microbiome of the lung based on age and gender [13].

Even less research has been done regarding the prevalence, etiology, and lung microbiome of patients with both bronchiectasis and HIV. One of the first descriptions of bronchiectasis in HIV patients was a case series published in 1992 [20], predating the availability of ART therapy. The exact etiology of bronchiectasis in HIV-positive patients remains unknown. It has been hypothesized that defective B lymphocyte and neutrophil function leads to decreased response to antigenic stimuli, which may predispose patients to increased lung infections that consequently act as a trigger for the above-mentioned cycle [21–23]. However, with the advent of newer antiretroviral therapy, there has been a decline in pulmonary infections due to an improved competency of the immune system.

HIV is now considered a chronic inflammatory disease that may have a different and even more direct role in the underlying pathogenesis of bronchiectasis [24]. A recent study on HIV-associated noninfectious lung disease suggests development of COPD in HIV patients due to a progressive loss of CD4 cells, which yields a persistent inflammatory state from CD8 T cell overexpression. It also suggests that HIV viral protein can exert direct effect on endothelial cells leading to pulmonary artery hypertension [25]. In this new age of understanding of the disease, there has been insufficient investigation regarding its association with bronchiectasis.

In our small sample, we noted that patients are routinely connected to the pulmonary service somewhat late in the course of their disease. Patients frequently did not receive adequate therapy and were often inappropriately treated for alternate and seemingly incorrect diagnoses.

As a result of these alternate diagnoses, patients routinely received broad-spectrum antimicrobial therapy that may have altered the respiratory flora. Instead of the typical organisms seen in patients with bronchiectasis such as Pseudomonas, nontuberculous mycobacterial and Aspergillus, we predominantly saw normal respiratory flora and colonizers, including Candida. Although our sputum samples were limited, there is research to suggest that the respiratory flora is altered in HIV-positive patients as compared to HIV-negative patients. The exact mechanism behind this finding is not understood [26].

Importantly, patients were uniformly not offered bronchodilator therapy for their bronchiectasis. They were diagnosed during an inpatient admission, and the results of their radiographic findings were not appropriately communicated to primary providers. As a result, referrals to the pulmonology clinic were rarely made. HIV-positive patients have been shown to have a higher independent risk of developing obstructive lung diseases [27], some of which may include bronchiectasis that is underrecognized. Referral to pulmonary subspecialists earlier in the clinical course may help to identify these patients sooner and provide them with more appropriate management.

The reason for lack of follow-through of care is hypothesized to be multifactorial. Beyond being high volume institutions with limited resources, both Kings County Hospital and Downstate Medical Center primarily serve the Central Brooklyn, East New York, and Flatbush populations; on average, these populations have lower education levels and higher poverty levels than those of New York City. Furthermore, there is a high percentage of uninsured patients [28]. All these factors contribute to limitations on health literacy, distrust of the system, and overall diminished access to care.

Another interesting observation to note in this study is that despite having access to almost 20 years of data from a very large cohort of HIV-positive patients, we were only able to identify these 14 patients with the concurrent diagnosis of bronchiectasis. While this may allude to a low prevalence of bronchiectasis in the HIV-positive population, given the underrecognition in our 14 patients, we instead posit that the prevalence of bronchiectasis in HIV patients may be grossly underestimated. This is understandable given its vague symptomatology that overlaps with other chronic pulmonary diseases. Unfortunately, many patients in our series were rehospitalized several times for vague respiratory symptoms that were thought to be due to an acute infectious process, but were more likely due to exacerbations of bronchiectasis. Improving recognition and management of bronchiectasis in our population could help diminish rehospitalization rates. It could also help to reduce the significant morbidity of repeated exposures to inappropriate therapy with antibiotics for presumed acute bacterial respiratory infections and instead allow focus on supportive therapy including bronchodilators and mucous clearance techniques. Better understanding the nature of bronchiectasis in the HIV-positive population can help to identify therapies better tailored to its more unique pathogenesis.

## 4. Conclusions

This case series highlights that bronchiectasis can carry a significant symptomatic burden in HIV-positive patients. With improvement in the life expectancy of HIV-positive patients, bronchiectasis will likely become a more important sequela of the disease that all primary providers and pulmonologists should be aware of and consider in their differential diagnosis for respiratory symptoms.

## Figures and Tables

**Table 1:** Patient characteristics and HIV/opportunistic infection history.

	Age	Gender	Congenital?	CD4	Viral load	Hx of PJP?	ARV	Prev tx of MAC?	Comorbidities
Pt 1	12	F	Yes	979	<50	No	TDF/FTC/ATV/r	No	None
Pt 2	17	F	Yes	4	68900	No	TDF/FTC/DRV/r	No	None
Pt 3	19	F	Yes	205	161	Yes	RAL/DRV/r	Yes	Esophageal/vaginal candidiasis, generalized seizures, anemia
Pt 4	22	F	No	6	40232	Yes	TDF/FTC/DTG	No	Squamous cell carcinoma in situ in skin, esophagitis, depression, oligomenorrhea, axillary abscess, seborrheic dermatitis, prurigo nodularis, postinflammatory hyperpigmentation
Pt 5	25	F	No	5	136	Yes	TDF/FTC/LPV/r	Yes	HIV-induced cardiomyopathy, CVA
Pt 6	35	F	No	715	5192	No	ETV/DRV/RAL	No	Asthma
Pt 7	41	F	No	2	1756	Yes	TDF/FTC/DRV/r	Yes	Aplastic anemia, recurrent HSV2 infections
Pt 8	48	M	No	797	29	No	TDF/FTC/RAL	Yes	CAD, BPH, pulmonary aspergillosis, metastatic rectal cancer
Pt 9	48	F	No	1	1942818	Yes	TDF/FTC/DTG	Yes	GERD, COPD, tobacco abuse, cocaine abuse, migraines, G6PD deficiency
Pt 10	54	F	No	319	N/A	No	ABC/EFV	No	Emphysema, HTN, marijuana use
Pt 11	55	F	No	333	40854	No	LPV/r/ATV	Yes	Stage IV metastatic cervical cancer s/p TAH (1999), recurrent UTI, HTN, hip replacement, osteoarthritis, CKD stage IV with nephrostomy tubes
Pt 12	62	F	No	211	<20	No	TDF/FTC/RPV	No	NHL (large B cell lymphoma s/p chemotherapy), hepatitis C, and substance abuse history on methadone, HTN
Pt 13	70	M	No	834	<48	No	ABC/AZT/LPV/r	Yes	COPD, DM
Pt 14	77	F	No	549	<20	Yes	AZT/3TC/DRV/r	No	HTN, HLD, CAD s/p PCI, left mastectomy (Ca buildup)

3TC: lamivudine; ABC: abacavir; AZT: zidovudine; FTC: emtricitabine; TDF: tenofovir; EFV: efavirenz; ETV: etravirine; RPV: rilpivirine; ATV: atazanavir; DRV: darunavir; LPV: lopinavir; r: ritonavir; RAL: raltegravir; DTG: dolutegravir.

**Table 2:** Distribution of bronchiectasis and microbiology data.

	Distribution of bronchiectasis	Sputum culture	PFT	Smoker?	Pack years
Pt 1	N/A	N/A	N/A	No	N/A
Pt 2	RML, lingula, B/L lower lobes	Candida albicans	N/A	No	N/A
Pt 3	RLL	Candida albicans	N/A	N/A	N/A
Pt 4	N/A	Normal throat flora	N/A	No	N/A
Pt 5	RU, RM, and B/L lower lobes	Normal throat flora	N/A	No	N/A
Pt 6	LLL, RLL, RUL, and RML, most severe in the RML	N/A	N/A	No	N/A
Pt 7	RML and inferior lingula	No growth	N/A	No	N/A
Pt 8	RUL, RML, RLL, LLL	Normal flora; Aspergillus (BAL)	Moderate obstructive	Yes	N/A
Pt 9	RU, RM, and B/L lower lobes	Candida	N/A	Yes	58
Pt 10	CT read does not specify distribution	N/A	N/A	Yes	15
Pt 11	RUL and LUL	Overgrown by non-AFB organisms	N/A	Yes	N/A
Pt 12	RUL and lingula	H. influenza, normal throat flora, overgrowth by non-AFB organisms	N/A	Yes	3
Pt 13	LUL, LLL	Morganella morganii, MRSA, strep viridans	Moderate obstructive	Yes	50
Pt 14	B/L lower lobe	N/A	N/A	No	N/A
